# Dengue Virus, Nepal

**DOI:** 10.3201/eid1403.070473

**Published:** 2008-03

**Authors:** Basu Dev Pandey, Kouichi Morita, Santa Raj Khanal, Tomohiko Takasaki, Isao Miyazaki, Tetsuro Ogawa, Shingo Inoue, Ichiro Kurane

**Affiliations:** * Tropical and Infectious Disease Hospital, Kathmandu, Nepal; † Nagasaki University, Nagasaki, Japan; ‡ Tribhuban University, Kathmandu, Nepal; § National Institute of Infectious Diseases, Tokyo, Japan; ¶ Pentax Company Limited, Tokyo, Japan

**Keywords:** Dengue virus, Nepal, mosquitoes, letter

**To the Editor:** *Dengue virus* belongs to the genus *Flavivirus,* family *Flaviviridae.* It has 4 serotypes: dengue virus type 1 (DENV-1), dengue virus type 2 (DENV-2), dengue virus type 3 (DENV-3), and dengue virus type 4 (DENV-4). Dengue virus is maintained in a cycle between humans and *Aedes aegypti,* domestic day-biting mosquitoes. Dengue virus induces clinical illness, which ranges from a nonspecific viral syndrome (dengue fever [DF]) to severe and fatal hemorrhagic disease (dengue hemorrhagic fever [DHF]). DF/DHF occurs primarily in tropical and subtropical areas of the world. Domestic dengue virus infection occurs in >100 countries; >2.5 billion persons live in these areas. Approximately 100 million cases of DF, 500,000 cases of DHF, and several thousand deaths occur annually worldwide (*1*). During the past decades, dengue virus has emerged in southern Asia; DF/DHF epidemics have occurred in Bhutan, India, Maldives, Bangladesh, and Pakistan (*2*–*4*).

From August through November 2006, the number of febrile patients increased in 4 major hospitals in the Terai region of Nepal: Nepalgunj Medical College, Bheri Zonal Hospital in Nepalgunj, Tribhuban Hospital in Dang, and Narayani subregional hospital in Birgunj. Patients with severe symptoms were referred to Sukraraj Tropical and Infectious Disease Hospital, Kathmandu, for diagnosis and treatment. The clinical features in most patients were consistent with signs of DF, but some patients showed signs (high fever, rash, ecchymosis, epistaxis, positive tourniquet test, liver dysfunction, and thrombocytopenia [platelet count <100,000/mm^3^]) consistent with the World Health Organization (WHO) definition of DHF. Ascites and plural effusion developed in 2 patients. Blood specimens were collected from all patients at the time of admission to the local hospitals. Particle agglutination (PA) assay (Pentax Ltd, Tokyo, Japan) (*5*) and immunoglobulin (Ig) M–capture ELISA (Dengue/JE IgM Combo ELISA kit, Panbio Ltd, Brisbane, Queensland, Australia) were performed. Dengue virus–specific IgM was detected in 11 patients who had fever, headache, and rash (Table). Each of these patients had negative results for Japanese encephalitis virus–specific IgM. Of the 11 patients, 10 had no history of travel to India or other dengue-endemic countries. DF or DHF was initially diagnosed for 7 patients, and viral encephalitis, typhoid fever, or viral fever was diagnosed for others without serologic tests. Reverse transcription–PCR and virus isolation were performed at Institute of Tropical Medicine, Nagasaki University, Nagasaki, Japan, but the dengue virus genome was not detected, and no virus was isolated, likely because sample collection was delayed and the sample was transported to Japan in a deteriorated condition.

**Table:** Table. Clinical and laboratory data for 11 patients admitted to hospitals and diagnosed with dengue fever or dengue hemorrhagic fever, Nepal, 2006*

Patient age, y/Sex	Month admitted	Location	Initial diagnosis	Travel history	Clinical signs and symptoms	Selected laboratory and other test results
20/M	Sep	Kathmandu	DF	Yes	Fever, headache, nausea	Hb 15.4 g/dL; TLC 10,500/mm^3^; Plt 185,000/mm^3^; blood culture for salmonellae negative; ALT 38 IU/L
27/F	Sep	Bardiya	Viral fever	No	Fever, headache, vomiting	TLC 5,600/mm^3^; blood culture for salmonellae negative
3/M	Sep	Salayan	Encephalitis	No	Fever, vomiting, convulsions	Widal negative; TLC 4,700/mm^3^
13/M	Oct	Sindhuli	Typhoid fever	No	Fever, headache	Widal negative; TLC 4,500/mm^3^; blood culture for salmonellae negative; *Brucella* antigen negative; chest radiograph normal
22/M	Oct	Birgunj	DHF	No	Fever, headache, vomiting, ascites	Bil 0.8 mg/dL; ALT 80 IU/L; Plt 22,000/mm^3^; chest radiograph normal
55/F	Oct	Dang	DF	No	Fever, headache, muscular pain	Plt 51,000/mm^3^; TLC 7,600/mm^3^; MP negative; ESR 20 mm/h; Bil 0.7 mg/dL
22/F	Oct	Birgunj	Viral fever	No	Fever, headache, body ache	*Brucella* negative; Widal negative; TLC 5,600/mm^3^
13/M	Nov	Dang	DF	No	Fever, headache, rashes	Plt 95,000/mm^3^; TLC 4,700/mm^3^; Hb 13.1 g%; Bil 0.8mg/dL; ALT 26 IU/L
35/F	Nov	Birgunj	DHF	No	Fever, headache, bruises; tourniquet: positive	Bil 0.81mg/dL; Plt 31,000/mm^3^; PT 2 min 30 s (control 14)
40/M	Nov	Birgunj	DF	No	Fever, headache, rashes	ALT 127IU/L; Plt 110,000 /mm^3^; PCV 38.8%;TLC 5,500/mm^3^; ultrasonography liver size, 16.8 cm
42/M	Nov	Dang	DF	No	Fever, headache, rashes	Bil 0.7 mg/dL; Widal test negative; TLC 6,800/mm^3^; Plt 164,000/mm^3^

*Blood specimens were collected at time of hospital admission. Diagnosis was confirmed by using immunoglobulin M–capture ELISA. DF, dengue fever; Hb, hemoglobulin;TLC, total leukocyte count; Plt, platelets; ALT, alanine aminotransferase; DHF, dengue hemorrhagic fever Bil, bilirubin; MP, malaria parasites; ESR, erythrocyte sedimentation rate; PT, prothrombin time; PCV, packed cell volume.

DF/DHF have been considered to be a possible public health threat to Nepal because DF/DHF epidemics have occurred recently in India and Pakistan, which reported several thousand cases and >100 deaths (*6*). The first DF case in Nepal was reported in 2004 (*7*). Further, the first DENV-2 strain of Nepal origin was isolated from a Japanese traveler who visited Nepal and in which DF developed after the patient returned to Japan. The isolated DENV-2 (GenBank accession no. AB194882) was 98% homologous with DENV-2 isolated in India (*8*). The prevalence of dengue virus antibody was reported to be 10.4% in the southwestern region of Nepal (*9*). These reports suggest that dengue virus has been circulating in Nepal for several years. Thus, DF/DHF has likely been misdiagnosed and illness caused by dengue virus underestimated in Nepal. In contrast, Japanese encephalitis has been a public health problem in southwestern region of Nepal, and large epidemics have occurred almost every year since 1978 (*10*). Nepal has no dengue surveillance programs, and health professionals do not usually consider dengue as a differential diagnosis.

The emergence occurred in the lowland Terai belt region, which borders the state of Bihar, India. The *Aedes* mosquito is known to persist in this region. The emerging DENV-2 is likely to have been introduced into Nepal from India.
